# Next Generation Sequencing Provides Rapid Access to the Genome of *Puccinia striiformis* f. sp. *tritici*, the Causal Agent of Wheat Stripe Rust

**DOI:** 10.1371/journal.pone.0024230

**Published:** 2011-08-31

**Authors:** Dario Cantu, Manjula Govindarajulu, Alex Kozik, Meinan Wang, Xianming Chen, Kenji K. Kojima, Jerzy Jurka, Richard W. Michelmore, Jorge Dubcovsky

**Affiliations:** 1 Department of Plant Sciences, University of California Davis, Davis, California, United States of America; 2 Genome Center, University of California Davis, Davis, California, United States of America; 3 Department of Plant Pathology, Washington State University, Pullman, Washington, United States of America; 4 Wheat Genetics, Quality, Physiology, and Disease Research Unit, United States Department of Agriculture-Agriculture Research Service (USDA-ARS), Pullman, Washington, United States of America; 5 Genetic Information Research Institute, Mountain View, California, United States of America; 6 Howard Hughes Medical Institute, Chevy Chase, Maryland, United States of America; 7 Gordon and Betty Moore Foundation, Palo Alto, California, United States of America; University of Nebraska, United States of America

## Abstract

**Background:**

The wheat stripe rust fungus (*Puccinia striiformis* f. sp. tritici, PST) is responsible for significant yield losses in wheat production worldwide. In spite of its economic importance, the PST genomic sequence is not currently available. Fortunately Next Generation Sequencing (NGS) has radically improved sequencing speed and efficiency with a great reduction in costs compared to traditional sequencing technologies. We used Illumina sequencing to rapidly access the genomic sequence of the highly virulent PST race 130 (PST-130).

**Methodology/Principal Findings:**

We obtained nearly 80 million high quality paired-end reads (>50x coverage) that were assembled into 29,178 contigs (64.8 Mb), which provide an estimated coverage of at least 88% of the PST genes and are available through GenBank. Extensive micro-synteny with the *Puccinia graminis* f. sp. tritici (PGTG) genome and high sequence similarity with annotated PGTG genes support the quality of the PST-130 contigs. We characterized the transposable elements present in the PST-130 contigs and using an ab initio gene prediction program we identified and tentatively annotated 22,815 putative coding sequences. We provide examples on the use of comparative approaches to improve gene annotation for both PST and PGTG and to identify candidate effectors. Finally, the assembled contigs provided an inventory of PST repetitive elements, which were annotated and deposited in Repbase.

**Conclusions/Significance:**

The assembly of the PST-130 genome and the predicted proteins provide useful resources to rapidly identify and clone PST genes and their regulatory regions. Although the automatic gene prediction has limitations, we show that a comparative genomics approach using multiple rust species can greatly improve the quality of gene annotation in these species. The PST-130 sequence will also be useful for comparative studies within PST as more races are sequenced. This study illustrates the power of NGS for rapid and efficient access to genomic sequence in non-model organisms.

## Abstract 2

(XLSX)

## Introduction

Wheat stripe rust caused by *Puccinia striiformis* Westend. f. sp. *tritici* Eriks. (PST), is one of the most devastating diseases of wheat worldwide [1], [2]. The dispersion of PST races with virulence for *Yr2* in the 1970s, *Yr9* in the 1990s, and *Yr27* in recent years contributed to large regional epidemics and crop losses [1], [3]. The appearances of new and more aggressive PST races at the beginning of the 21^st^ century [1], [4] pose an increasing global threat to wheat production [2], [3]. In the US alone, epidemics caused by these post-2000 races resulted in average yield losses of $156 million dollars per year between the years 2000 and 2005. In the last two years these more virulent races expanded and devastated major wheat-producing areas in China, northern and eastern Africa, western and central Asia, and the Middle East [1], [2] (www.globalrust.org).

The understanding of pathogenicity and virulence factors, and of their evolution, is critical to the development of more effective breeding strategies for durable resistance. However, progress in these areas has been hampered by the lack of PST genome sequence information. The genome sequence of *P. graminis* f. sp. *tritici* (PGTG; causal agent of wheat stem rust) has recently been published [5] and the genome of *P. triticina* (PTTG; causal agent of wheat leaf rust) is currently being fully sequenced and annotated (http://www.broadinstitute.org/annotation/genome/puccinia_group/Info.html). However, a genome sequence for PST is not currently available (a project is in progress at the Broad Institute to sequence and annotate race PST-78). Only a limited number of EST resources (<3,000) are currently available for PST [6], [7], [8].

Next generation sequencing (NGS) is a powerful tool that has provided dramatic improvement in sequencing speed and depth together with a steep decline in associated costs compared to previous sequencing technologies. Today, NGS is less expensive, quicker and more efficient to access gene sequences by whole genomic sequencing than traditional gene-by-gene approaches. The application of NGS in plant pathology and in plant-microbe interaction research promises to shorten the overall time for development of molecular genetic information necessary for functional and translational studies. Here we report sequence information for a large part of the PST race PST-130 genome obtained by Illumina sequencing as an example of rapid and efficient genome characterization in a non-model species with little prior molecular information.

## Results

### Illumina sequencing and *de novo* contig assembly

The sequence reads from race PST-130 together with the resulting assemblies are available from NCBI (Whole Genome Shotgun project: AEEW00000000; Sequence Read Archive: SRP002642). The raw and trimmed reads are summarized in Table 1, together with the GenBank accession numbers. After trimming low quality regions and excluding low quality reads (see Material and Methods section), a final set of 79,156,610 reads was obtained for a total length of 6,708,133,571 nt. A total of 29,307 contigs were assembled with a total length of 65,398,578 nt using CLC Genomic Workbench v4.0.

**Table 1:** Summary of raw and trimmed reads and assemblies of PST-130 genomic DNA.

Sequencing run a	GenBank accession number	No. of cycles	No. ofpaired-endreads b	Filtered reads			
				Total No. b	Length (nt)		
					Total	Average	Median
**PST-130-1A**	SRX022476	85	27,999,777	18,671,826	1,364,151,052	73.1	79
**PST-130-1B**	SRX022476				1,469,928,965	78.7	85
**PST-130-2A**	SRX022218	101	25,809,472	20,906,479	1,943,054,211	92.9	101
**PST-130-2B**	SRX022218				1,930,999,343	92.4	101
**Assembly**	AEEW00000000 c			29,178	64,782,816	2,220	900 ^d^

a 1 and 2 indicate different Illumina sequencing runs, and A and B indicate paired-end reads.

b The number of paired-end reads needs to be multiplied by 2 to obtain the total number of reads.

c The following 22 contigs (84 kb) include PST-130 mitochondrial DNA: PST130_238, PST130_239, PST130_275, PST130_6617, PST130_6630, PST130_6699, PST130_6903. PST130_7099, PST130_7122, PST130_7145, PST130_7255, PST130_10273, PST130_10278, PST130_20206, PST130_20452, PST130_20455, PST130_20456, PST130_20466, PST130_20619, PST130_28223, PST130_28226, PST130_28239.

c N50 = 5,137 nucleotides.

In order to identify DNA sequences resulting from contamination (e.g. aphids, bacteria, plant cells, etc.) during spore collection from infected wheat plants we queried the PST-130 assemblies against the GenBank nt database (ftp.ncbi.nih.gov/blast/db/FASTA/nt.gz; 15 July 2010), 30,696 wheat contigs (37.8 Mb) assembled from a 454 transcriptome study of hexaploid wheat flag leaves [9], the complete *Triticeae* Repeat Sequence Database (TREP; http://wheat.pw.usda.gov/ITMI/Repeats) and the 454 sequence reads of the hexaploid wheat genome (http://www.cerealsdb.uk.net/). One hundred and twenty nine contigs showed high similarity (BLASTN, % identity ≥90%; E-value ≤e^−10^; low complexity filter “on”) to sequences from *Viridiplanta*e (74 contigs), *Metazoa* (54 contigs) and *Bacteria* (1 contig); these were excluded from further analysis and not submitted to GenBank. Assemblies with higher similarity to PGTG and PTTG sequences (www.broadinstitute.org) were retained. The remaining 29,178 contigs with a length of 64,782,816 nt were submitted to GenBank WGS division (Table 1). The N50 of the submitted contigs was 5,137 nt, indicating that 50% of the entire assembly is contained in contigs equal to or larger than this value. Based on similarity to PGTG mitochondrial DNA (BLASTN, % identity ≥90%; E-value ≤e^−10^), 22 PST-130 contigs (see footnote Table 1), accounting for a total of 84 kb, are likely to be part of the PST mitochondrial genome.

To our knowledge, there are no estimates of PST genome size and therefore it is not possible to calculate the genome coverage of the assembled PST-130 contigs precisely. Assuming that the PST genome is not larger than the PGTG genome (88.6 Mb; [5]), the 6,708 Mb of trimmed PST sequences generated in this study should provide at least 75 x genome coverage. To provide an independent estimate of coverage, we selected 10 single copy genes (18 kb total genomic sequence) and determined the number of Illumina reads that mapped to them using the Bowtie aligner (v0.12.7 [10]). Single copy genes were identified as regions in our contig dataset showing a unique significant TBLASTN match to PGTG rust proteins (best match E-value ≤e^−10^ and second best match E-value >0.1). The PST-130 reads mapped to the single copy genes provided a 55±2 x coverage when no mismatches were allowed (Bowtie parameters: -v 0 --best) and a 62±2 x coverage when one mismatch was allowed in the global alignment (Bowtie parameters: -v 1 --best). Even with the lowest estimated coverage of 55 x, the probability of recovering any PST region accessible to Illumina sequencing is close to 1.00 (see Material and Methods section).

**Figure 1:**
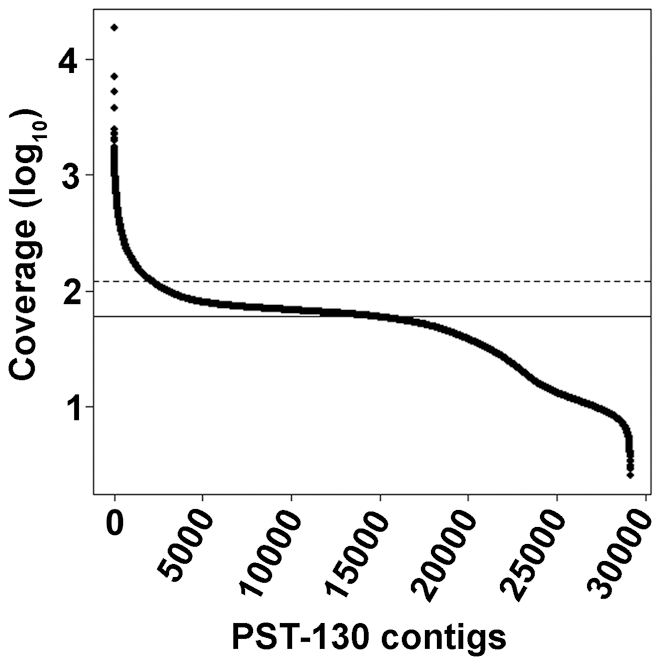
Coverage distribution by contig (log_10_ scale). Contigs are ordered by coverage (calculated using Bowtie [10]). Some repetitive regions are assembled into single contigs resulting in higher coverage and an underestimation of the genome size. The horizontal line indicates the median coverage (60.4x) and the dotted line the 2-fold coverage (120.8x) used as the threshold to calculate the proportion of the genome represented by contigs assembled from duplicated regions.

A large proportion of the PST-130 reads (95%) was assembled into contigs (64.8 Mb). Based on this proportion, the PST nuclear genome size (100%) can be estimated to be at least 68.2 Mb. However, this number underestimates the true genome size because sequences from similar repeats are assembled into common contigs by the CLC Genomic Workbench program (Figure 1). To estimate the size of this repetitive fraction, we identified contigs with coverage higher than twice the median coverage (>120.8 x excluding mitochondrial sequences, Figure 1) and multiplied the contig size by the fold-coverage (coverage/ median coverage; Material S1). This calculation suggests that an additional 10.6 Mb should be added to the initial 68.2 Mb, resulting in an estimated size of the PST-130 genome of 78.8 Mb, 11% smaller than the current estimate of the PGTG genome [5].

To provide an estimate of the proportion of the genes represented in the 64.8 Mb PST assembly, we compared it with the 2,848 PST ESTs sequences available in GenBank (1,487 from haustoria [6] and 1,361 from urediniospores [7], [8]). To refine this estimate, we first verified whether there were potential contaminant sequences within the PST ESTs in GenBank. Among the 549 PST ESTs that did not match our PST-130 assembly, 237 ESTs sequences showed significant similarity (BLASTN, E-value < e-10) to sequences from *Viridiplantae* (161 ESTs), *Metazoa* (11 ESTs), or *Bacteria* (3 ESTs) in GenBank nt database and to 62 wheat ESTs in GenBank EST database. After excluding these potential 237 contaminant sequences, 88% of the remaining 2,611 PST ESTs showed high similarity to our PST-130 assembly (BLASTN, >90% identity at the DNA level; E-value < e^−10^; median aligned length of ESTs: 86.7%). However, the proportion of genes represented in the PST-130 assembly may be even higher than 88% since none of the EST sequences used in this comparison were from race PST-130. Race-specific differences could be partially responsible for the lack of detection of some of the remaining 312 ESTs (12% of 2,611). In addition, some of the 312 non-detected ESTs might be undetected contaminants since only 37 of them showed significant similarities to predicted proteins from PGTG or PTTG (www.broadinstitute.org [5]; BLASTX; E-value < e^−10^).

To assess the quality of the coding sequence assemblies in the PST-130 contigs, we performed similarity searches using predicted peptides present in the PGTG genome (TBLASTN, E-value ≤e^−10^). This comparison showed that of the 20,566 predicted peptides of the PGTG genome [5] 13,072 were similar to sequences predicted from the PST-130 contigs. Of these alignments, 84% cover more than 50% of the length of the PGTG peptides (mean length coverage ±SD: 76.4% ±22.5). We also compared our contigs with a set of 458 highly conserved protein families that occur in a wide range of eukaryotic organisms [11]. Ninety percent of these core eukaryotic genes showed significantly similarities with proteins predicted from the PST-130 contigs (TBLASTN, E-value < e^−10^) suggesting that reads were correctly assembled and confirming a comprehensive genome coverage. Of these alignments, 92% cover more than 50% of the length of the conserved peptides (mean length coverage ±SD: 75.9% ±22.4).

### Microsynteny between PST-130 and PGTG

**Figure 2:**
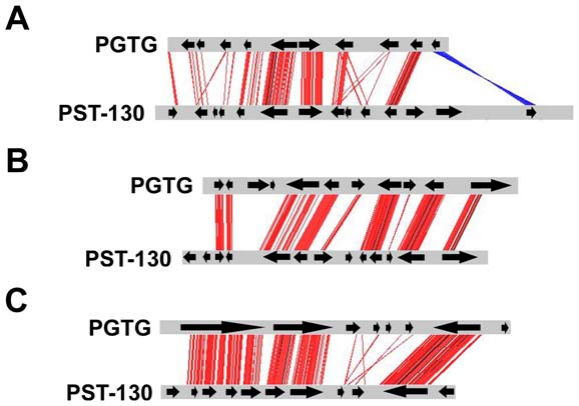
Microsynteny between PST-130 and PGTG contigs. Microsynteny between PGTG [AAWC01001263 (A), AAWC01003253 (B), AAWC01000559 (C) [5]] and PST-130 (PST130_8308, PST130_8617, PST130_7101) contigs. Contig alignment and similarity visualization was done using ACT-Artemis Comparison Tool (http://www.sanger.ac.uk/resources/software/act/). Red and blue lines indicate similar regions between PST-130 and PGTG contigs (% identity ≥60). Blue lines indicate inversions. Arrows correspond to putative genes predicted by geneid in PST-130 contigs and annotated genes in PGTG contigs [5].

We evaluated the level of micro-synteny between PST-130 and PGTG by comparing the composition of the genes in the 20 largest PST contigs with the annotated gene order in the PGTG genome (www.broadinstitute.org [5]; putative orthology based on BLASTX, E-value ≤e^−10^). Figure 2 graphically shows the colinearity of three PST-130 contigs with their corresponding orthologous PGTG contigs. Most of the 20 largest PST-130 contigs have long stretches that are colinear to single PGTG contigs (average ± SE: 56% ±5). In most cases when multiple PGTG contigs corresponded to a single PST contig, they belonged to the same PGTG supercontig, supporting the accuracy of the PST-130 assemblies (Table S1). Most of the corresponding PGTG proteins that matched a PST contig had consecutive or very close coordinates, also indicating extensive microsynteny between PST and PGTG genomes. The microsyntenic segments were frequently interrupted by non-colinear genes, and by altered order of genes, suggesting the existence of multiple rearrangements, insertions and deletions during the evolution of these species.

### Transposable elements in PST-130 contigs

We identified over 140 families of autonomous and non-autonomous transposable elements (TEs) that represent 17.8% of the PST-130 contig sequences (Table 2). The percent contribution of TEs to the entire PST genome is likely larger because sequences from similar repeats are assembled into common contigs. The most abundant TEs are DNA transposons (8.2%) and LTR retrotransposons (7.2%) with all other classes representing only 2.4% of the contigs' length. The relative abundance of major superfamilies within the major TE classes is described in Table 2. All classes and superfamilies of TEs identified in the PST genome are also represented in the *P. graminis* f. sp. *tritici* (PGTG) genome [5]. Representative TE sequences from the PST and the PGTG genome were annotated and deposited in Repbase [12].

**Table 2:** Summary of repeat contents in the PST-130 contigs.

Transposable element class	Contig coverage(% assembled length a)	Major Superfamilies(% within class)	
DNA transposons b	8.2		
		*hAT*	(13.6)
		*Harbinger*	(10.3)
		*Mariner/Tc1*	(9.1)
		*MuDR*	(6.7)
		*EnSpm*	(6.4)
		*Helitron*	(5.0)
		*P*	(1.2)
LTR retrotransposons	7.2		
		*Gypsy*	(52.1)
		*Copia*	(32.3)
		*DIRS*	(5.3)
LINEs	1.0		
		*Tad1*	(18.6)
SINEs	0.01		
			
Satellites and microsatellites	0.1		
Unclassified repeats	1.3		
Total	17.8		

a Total length of assembled contigs  = 64.8 Mb.

b The remaining DNA transposons (48%) are represented by ∼60 families of non-autonomous elements which could not be assigned to any specific superfamilies.

A phylogenetic analysis of *Harbinger* elements using the conserved DDE-transposase (∼200 amino acids) revealed an unexpectedly high level of similarity between rust and plant *Harbinger* elements (average % identity ±SD: 54.6±3.6) relative to the long divergence time between their respective two evolutionary lineages (∼1,500 million years, [13]). A phylogenetic analysis (Figure S1) revealed a well defined cluster (bootstrap 99%) including *Harbinger* elements from rusts (*Puccinia* and *Melampsora*, Dikarya; Basidiomycota), plants, and *Phytophthora infestans* (causal agent of potato blight, Stramenopiles; Oomycetes), that was separated from the cluster including *Harbinger* elements from other fungi (*Talaromyces stipitatus* and *Tuber melanosporum,* Dikarya; Ascomycota), animals and protists (Figure S1). An ancient horizontal transfer of *Harbinger* elements between rust and plants is the most parsimonious explanation for these results, but we cannot rule out more complex scenarios combining differential evolutionary rates and differential deletions of *Harbinger* groups in different evolutionary lineages.

Detailed analysis of the TE in the sequenced rust species revealed several unusual elements including TEs from the *P* superfamily (Repbase ID *P-1_PSt)*, previously unknown in any Dikarya [14]. Although no intact *P* elements were found in the two *Puccinia* genomes, we found them in the recently sequenced *Melampsora larici-populina* genome [5]. Two autonomous and two non-autonomous *P* elements from the *M. larici-populina* genome flanked by characteristic 8-bp target site duplications (TSD) were deposited in Repbase (IDs: *P-1_MLP*, *P-2_MLP*, *P-N1_MLP* and *P-N2_MLP*).

We also identified a distant branch of DNA transposons belonging to the *MuDR* superfamily named *MuDRF* (e.g. Repbase ID *MuDRF-1_PSt*, *MuDRF-2_PSt* and *MuDRF-3_PSt*). *MuDRF* elements encode one protein that includes a GCM (glial cell missing) DNA-binding domain specific for *MuDRF* elements (Figure S2) and a DDE-transposase domain.

Finally, we identified *Sagan* elements, which share TA target site duplications and short terminal inverted repeats with all *Mariner/Tc1/IS630* elements, but represent a more divergent group. *Mariner/Tc1/IS630* superfamily includes at least three diverse branches: *Mariner, Tc1* and *Pogo*. *Sagan* elements differ from those in the previous three groups by a long insertion in their DDE-transposases, between D and E residues in the conserved DDE triad. *Sagan* elements were found in various fungi including Basidiomycota, Ascomycota (*T. melanosporum*), Mucoromycotina (*R. oryzae*), as well as in stramenopiles (*Albugo laibachii* and *Aureococcus anophagefferens*), and alveolates (*Perkinsus marinus*).

### Gene discovery

To facilitate the rapid identification of genes within the PST-130 genome we used the *ab initio* gene prediction program geneid (genome.crg.es/software/geneid/) with parameters trained for PGTG. We identified 22,815 putative coding sequences (CDS) encoding predicted proteins of an average length of 277 amino acids (Material S2). From these predicted proteins we eliminated 2,392 associated with TEs by similarity searches to Repbase (BLASTP, E-value ≤e^−5^). The final set of 20,423 predicted proteins is presented in Material S3 and includes 6,254 pfam accession numbers and names (http://pfam.sanger.ac.uk/), 2,200 GO descriptions (http://www.blast2go.org/) and the most similar PGTG protein accessions and annotations (BLASTP, E-value ≤e^−5^).

**Figure 3:**
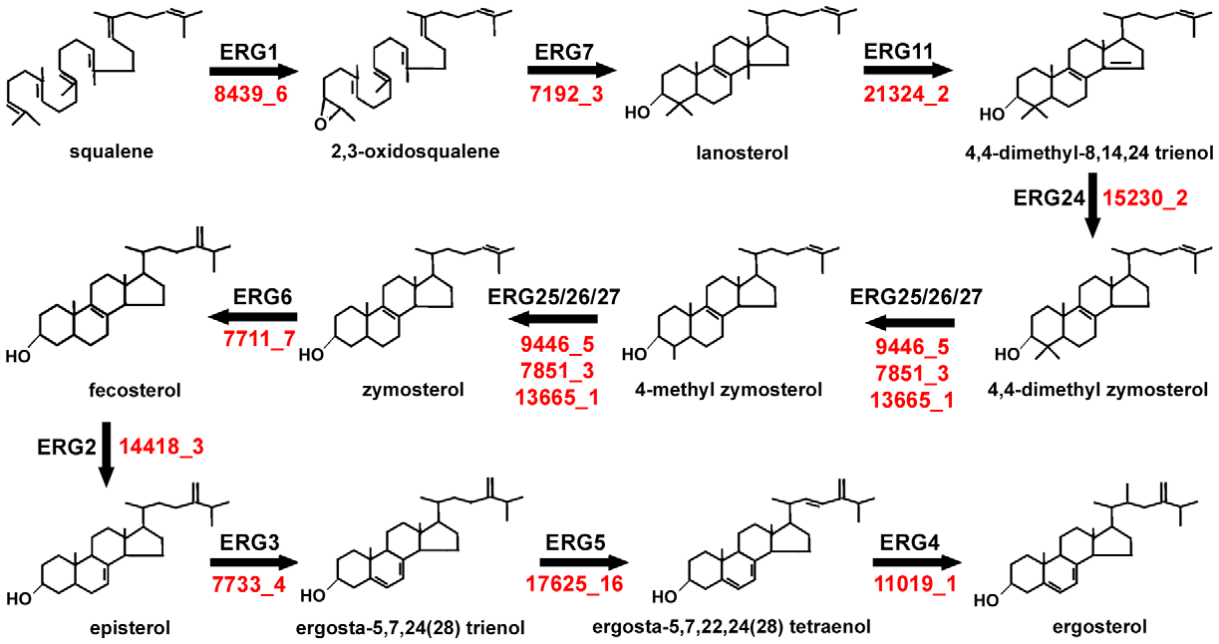
Genes identified in the PST-130 contigs that encode proteins involved in the ergosterol biosynthetic pathway. In red the IDs of the PST-130 manually curated genes. ERG1: squalene epoxidase (HQ698552); ERG2: C-8 sterol isomerase (HQ698553); ERG3: C-5 sterol desaturase (JN033203); ERG4: C-24 sterol reductase (partial clone, not submitted to GenBank); ERG5: C-22 sterol desaturase (JN033204); ERG6: C-24 sterol methyl-transferase (JN033205); ERG7: lanosterol synthase (JN033206); ERG11: lanosterol 14α-demethylase (HQ698554); ERG24: C-14 sterol reductase (HQ698555); ERG25: C-4 sterol methyloxidase (JN033207); ERG26: C-4 sterol decarboxylase (JN033208); ERG27: C-3 sterol ketoreductase (JN033209).

Since this set of predicted proteins is based exclusively on *in silico* predictions from a single program, it should be used with caution, and with the understanding that some of these predictions need further curation and validation. We used two approaches to estimate the quality of the geneid predictions. First, we compared the 20,423 predicted proteins with a set of 458 highly conserved protein families [11]. This comparison showed that geneid predictions covered on average 84% of the length of the conserved proteins. In the second approach, we compared 20 geneid models with the corresponding PGTG models ([5] Table S2). The selected genes included the twelve genes involved in the ergosterol biosynthetic pathway (Figure 3), two genes encoding an Argonaute and a RNAse III, and six random ones from a list of genes with significant similarity (BLASTP, E-value ≤e^−10^) among PST and PGTG. The ergosterol pathway was selected because of its importance as targets of fungicide products, whereas the Argonaute and RNaseIII were selected to confirm the presence of the RNAi pathway in PST, which is involved in recently proposed resistance strategies [15]. The percentage of geneid correctly predicted amino acids relative to the 20 PST annotations curated by comparative genomics was 74.9%. Similar percentages (81%) of correctly predicted amino acids were obtained when the corresponding curated PGTG annotations were compared with available predicted gene models [5].

The PST-PGTG comparative genomics approach showed that a large proportion of prediction errors occurred at the borders of the exon – intron junctions. These junctions were easy to detect in the comparative analysis because PST and PGTG exhibit a high level of identity among predicted proteins (>78% identity in this set) and almost no DNA sequence conservation in the intronic and intergenic regions. In all cases, when an exon or exon border from a predicted protein showed no similarity to the other rust species, it was possible to find alternative annotations that resulted in protein identities higher than 78% in the re-annotated region. The manually curated PST-130 genes were deposited in GenBank (accessions HQ698552-HQ698561 and JN033203- JN033211). The curated PGTG protein sequences are included in Text S1.

### PST-130 predicted secretome

Microbial plant pathogens secrete effector proteins, some of which subvert the plant innate immune response and enable infection [16]. In spite of their importance to our understanding of the stripe rust – wheat pathosystem, no wheat stripe rust effector has been validated so far. To produce a preliminary catalogue of potential PST effectors the geneid predicted PST-130 proteins were analyzed using a bioinformatics pipeline for secreted proteins similar to the ones used in *Phytophthora* [17] and PGTG [5]. First, we tested for the presence of a signal peptide using the SignalP V3.0 software [18]. The 1,188 sequences with a signal peptide (HMM Sprob ≥0.9; average cleavage site position: 24±9) were further filtered with the TM-HMM program to remove transmembrane proteins (transmembrane domain length ≥18 aa and not in the first 60 aa [19]) and with the TargetP program to exclude mitochondrial proteins (RC1 or RC2 [20]). Twenty three and seventy seven sequences were removed by TM-HMM and TargetP filtering, respectively. Using this pipeline we identified 1,088 coding sequences predicted to encode secreted PST-130 proteins with median length of 165 amino acids (Material S4). When the same pipeline was applied to the 20,566 PGTG predicted proteins [5] 1,463 potential effector proteins were identified.

Of the 1,088 PST potentially secreted proteins, 730 (67%) were similar to PGTG sequences, but only 293 (27%) shared similarity with the set of 1,463 PGTG sequences selected as potential members of the PGTG secretome (BLASTP, E-value ≤e^−5^ Material S4). This proportion is lower than the 69% of PST proteins that are significantly similar to PGTG proteins using the same parameters (Material S3), consistent with the hypothesis that sequences involved in pathogenesis evolve at higher rate than other proteins [5]. This low conservation among candidate effectors is also reflected in the small proportion of sequences (4%) that had significant similarities to sequences in the GenBank nr database (BLASTP, E-value≤e^−6^; Material S4) and in the lack of PST-130 or PGTG homologues to the known effectors *AvrM*, *AvrL567*, *AvrP123*, and *AvrP4* from *Melampsora lini* (proteome comparisons, BLASTP E-value ≤e^−3^, and translated contig comparisons, TBLASTN E-value ≤e^−3^; [5]). This set of 1,088 potentially secreted proteins is larger than the numbers of candidate effectors identified in other species [16] and undoubtedly contains proteins with other functions. However, it provides a tractable number of proteins that can be analyzed further for effector activity.

Twenty five of the candidate PST effectors share similarity with 27 PGTG proteins included in the 100 most highly up-regulated genes in infected wheat tissue compared to resting urediniospores (BLASTP, E-value ≤e^−10^; [5]). Notably, among these 25 proteins is the predicted protein PST130_10308_4 (75% similar to PGTG_17547; BLASTP, E-value = 2e^−52^), reported to be the most highly expressed gene in the PGTG haustoria, but absent outside of the *Pucciniales* clade [5]. Other interesting candidates among the 1,088 predicted PST effectors include five PST proteins with significant BLASTP similarity to the secreted protein RTP1 from *Uromyces fabae* [21], namely PST130_7686_8 (E-value = 2e^−47^), PST130_10165_7 (8e^−27^), PST130_10165_4 (3e^−22^), PST130_10165_6 (4e^−17^), and PST130_10209_2 (4e^−14^).

A cluster analysis of the 1,088 PST candidate effectors grouped 309 PST proteins into 116 groups (range 2-16, average ± SD: 2.7±1.8 candidate effectors per group), while the remaining 779 proteins showed no significant similarity to other members in the dataset (BLASTP, E-value ≤e^−10^). All 16 members of the largest cluster contained the *N*-terminal [Y/F/W]xC motif (100% FxC; Figure S3), which is associated with secreted proteins in *Blumeria graminis* and other rust species including PGTG and PTTG [22]. The same motif was observed in 121 (11%) of the 1,088 candidate PST effectors (within the first 17 amino acids after the predicted cleavage site), a significant enrichment compared to the 3% frequency of this site in the overall predicted PST-130 proteome (Material S4). In the majority of the proteins (101), the first amino acid of the motif is a phenylalanine (FxC) as observed in PGTG and PTTG proteins, but not in *B. graminis* where the predominant starting amino acid is a tyrosine (YxC [22]). In addition, the three largest clusters of PST-130 candidate effectors were also characterized by conserved cysteine (C) residues with other eukaryotic *Avr* genes (Figure S3).

Variants of the RxLR motif were recently shown to be sufficient for delivery of some fungal effectors into host cells [23]. Among the 1,088 candidate PST effectors, we identified 72 proteins containing these related motifs within the first 100 N-terminal amino acids after the predicted cleavage site (Material S4). Of these 72 proteins 15 were similar to candidate secreted PGTG proteins (BLASTP, E-value≤1e^−10^; Material S4).

## Discussion

This 64.8 Mb *de novo* assembly of the stripe rust race PST-130 provides a first view of the genome of this economically important wheat pathogen. This information is a useful resource to identify and mask repetitive PST sequences, to rapidly clone PST genes, and to access their promoters and other potential regulatory regions (e.g. for genomic tools such as gene capture that require genomic sequences). The geneid *ab initio* predictions and annotation of 20,423 proteins (69% of them similar to PGTG proteins) provide an alternative entry point to identify genes within the PST genome. This information fills a gap in our knowledge of this pathogen, for which only limited sequence information was previously available. Although the PST-130 contigs and predicted proteins described here provide access to most of the PST genes (by BLASTN and BLASTP searches, respectively), they do not replace the need for a high quality, annotated reference genome of PST for more comprehensive genomic studies.

We showed before in rice and barley that a comparative genomics approach can be used to improve the annotation of *ab initio* predicted genes [24]. These grass species showed similar levels of divergence in coding and intronic regions to those reported here between the PST and PGTG genomes. The annotation of 15 out of 20 geneid predicted PST genes was improved by manually curating them using this comparative genomics approach. The high level of protein identity observed between PST and PGTG (average identity ± SD 78% ±5 in the 20 tested genes), together with the high level of sequence divergence observed in the intronic and intergenic regions, increase the power of this comparative genomics approach and provide an effective strategy to improve gene models generated by *ab initio* gene prediction programs in the wheat rust species.

In addition to a relatively high degree of identity between the PST-130 and PGTG proteins, we detected extended regions of micro-synteny between the genomes of these two species (Figure 2; Table S1). More than half of the length of the longest PST contigs showed colinearity with regions of the annotated PGTG genome, facilitating the identification of orthologous genes. A more complete analysis including macro-synteny comparisons among PST and PGTG chromosomes requires the assembly of complete genomes from both species.

The PST-130 sequence provided here is also expected to be useful for comparative studies within PST once the annotated, high quality reference sequence of PST-78 becomes available. This comparison would be particularly interesting because these two races are representative of the more virulent races that have appeared since 2000 [1], [25] and that are responsible for the current global epidemic of stripe rust [2], [4]. PST-130, which was first detected seven years after the discovery of PST-78, has all the virulences known to be present in PST-78 as well as additional virulences to the differential cultivars Moro (*Yr10*, *YrMor*), Produra (*YrPr1*, *YrPr2*), and Stephens (*Yr3a*, *YrSt1*, *YrSt2*) [25]. Therefore, the comparative sequence analysis of these races may provide some candidates genes for the effectors recognized by these resistance genes. Next-generation sequencing of multiple PST races (and their rapid public release) will greatly accelerate current efforts to understand and control this devastating wheat pathogen.

## Materials and Methods

*Puccinia striiformis* f. sp. *tritici* race PST-130 was first isolated in Oregon and Washington in 2007 and was characterized using a set of 20 wheat differentials [25]. PST-130 urediniospores were collected from infected wheat plants and dried in a desiccator at 4°C for five days and then stored in aluminum foil bags in liquid nitrogen. DNA was extracted from dried urediniospores using the CTAB method as described by Chen et al. [26].

A single DNA library was prepared from 5 µg of total genomic DNA that was randomly fragmented using the Bioruptor sonicator (Diagenode). The library was prepared with the NEB Next DNA Sample Prep Master Mix according to manufacturer's instructions (New England Biolabs, MA USA). Size selection was performed after adapter ligation for 500 bp fragments. The fragments were purified using the Nucleotrap kit (Clontech) and eluted in 30 ul elution buffer and PCR-enriched using 14 PCR cycles. Paired end adapters and PCR primers 1.0 and 2.0 were obtained from Illumina. Library quality and quantity were confirmed before sequencing using a Bioanalyzer (Agilent).

Sequencing was done on an Illumina Genome Analyzer II at the DNA Technologies Service core at UC Davis (http://genomecenter.ucdavis.edu). Two paired-end sequencing runs were carried out, the first using 85 cycles (PST-130-1) and the second using 101 cycles (PST-130-2). Low quality sequences were removed using custom scripts available from http://code.google.com/p/atgc-illumina/. The primary output of the Illumina pipeline (qseq files) was used to extract high quality sequences. The parser, http://code.google.com/p/atgc-illumina/wiki/Illumina_QSEQ_Parser, analyzed quality scores in qseq files and trimmed all the nucleotides after the first failed score (‘B’ score; see http://code.google.com/p/atgc-illumina/wiki/Illumina_Quality_Scores).Upon trimming, sequences shorter than 40 nt and those where GC content was not within the 20%–80% range were excluded. FASTA files with high-quality trimmed sequences were used for downstream analysis.

The trimmed and filtered reads were then assembled using CLC Genomic Workbench 4.0 software (http://www.clcbio.com/). The following parameters were applied: mismatch, insert, and deletion cost  = 3; length fraction  = 0.3; similarity  = 1.0 no global alignment; conflict resolution  =  vote; ignore nonspecific matches; min contig length  = 300 bp; paired-end distance  = 100–600 bp.

Assemblies were deposited at GenBank under accession AEEW00000000. BLAST analysis was run locally using BLAST 2.2.21 (NCBI). PST ESTs were obtained from GenBank. Sequences of PGTG, PTTG and *M. larici-populina* were obtained from the Broad Institute (www.broadinstitute.org [5]) and from the DOE Joint Genome Institute (genome.jgi-psf.org [5]) and were used to test the presence of contaminant sequences in our assemblies and for comparisons of the predicted PST-130 gene models. Micro-colinearity analysis was done by parsing the coordinates of PST-130 and PGTG orthologous sequences identified by similarity searches using TBLASTN (E-value<e^−10^) with PGTG proteins as search queries and PST-130 and PGTG contigs as databases. BLAST and SAM format alignment results generated by Bowtie were parsed with custom Python, Perl and Unix shell scripts that are all available upon request.

The probability of recovering any PST-130 region accessible to Illumina sequencing was calculated using a formula derived from the one proposed by Clarke and Carbon [27]: P = 1 - (1 - W/N)N, where N =  number of fragments and W =  genome coverage.

The composition of transposable elements was determined by CENSOR [28] using a database of manually annotated libraries of repeats from PST-130, PGTG and *M. larici-populina* [5], combined with the remaining Repbase entries [12]. Exon-intron structures of transposable elements were predicted with the aid of Softberry FGENESH (linux1.softberry.com). Protein sequences of transposable elements were aligned using MAFFT with the linsi option [29]. Maximum likelihood tree was constructed at the PhyML 3.0 server (www.atgc-montpellier.fr/phyml/) [30] with 100 bootstrap replicates for the amino acid substitution model LG.

## Supporting Information

Figure S1 **Phylogeny of elements of the** ***Harbinger*** **superfamily of DNA-transposons.** The conserved DDE-transposase domain (∼200 aa) was used for phylogenetic analysis using the maximum likelihood algorithm. The tree was rooted using the outgroup *ISL2EU* and the numbers at nodes are bootstrap values of 100 replicates (only values >50% are shown). *Harbinger* elements from rust are colored in purple, those from *Phytophthora infestans* are in red, and those from plants are in green. All sequences used are deposited in Repbase. The elements were obtained from the following species: Fungi [*Puccinia striiformis* f. sp. *tritici* (PSt), *Puccinia graminis* (PGr), *Melampsora larici-populina* (MLP, Mlarici), *Allomyces macrogynus* (AllMac), *Ascosphaera apis* (AAp), *Phycomyces blakesleeanus* (PB), *Pleurotus ostreatus* (PleOst), *Talaromyces stipitatus* (TSt), *Tuber melanosporum* (TMe)]; Plants [*Arabidopsis lyrata* (ALy), *Fragaria vesca* (FV), *Malus x domestica* (Mad), *Medicago truncatula* (Mt), *Oryza sativa* (OS), *Populus trichocarpa* (PTr), *Selaginella moellendorffii* (Smoe), *Sorghum bicolor* (SBi), *Triticum aestivum* (TA), *Vitis vinifera* (VV), *Zea mays* (ZM)]; Oomycetes [*Phytophthora infestans* (PI)]; Animals [*Aedes aegypti* (AA, AAe), *Anopheles gambiae* (AG), *Branchiostoma floridae* (BF), *Ciona savignyi* (Cis), *Danio rerio* (DR), *Drosophila willistoni* (DW), *Drosophila yakuba* (DYa), *Gasterosteus aculeatus* (GA), *Hydra magnipapillata* (HM), *Nematostella vectensis* (NV), *Strongylocentrotus purpuratus* (SP), *Xenopus tropicalis* (XT)]; Protists [*Ectocarpus siliculosus* (ES), *Emiliania huxleyi* (EmiHux), *Monosiga brevicollis* (MBr), *Naegleria gruberi* (Ngru), *Thalassiosira pseudonana* (TP), *Trichomonas vaginalis* (TV)].
(PDF)

Figure S2 **Alignment of GCM domains coded by glial cells missing (GCM) genes and by** ***MuDRF*** **transposable elements.** Similarity to other *MuDR* transposases was established after four PSI-BLAST iterations using *MuDRF* transposase as a query. *MuDRF* elements were also found in other Basidiomycota, including PGTG, *M. larici-populina*, *Laccaria bicolor*, *Schizophyllum commune* and *Coprinopsis cinerea*. Outside fungi we also identified a full-length *MuDRF* element in *Heterolobosea (Naegleria gruberi). MuDRF* elements are flanked by 9-bp TSD, characteristic for *MuDR* elements. Triangles indicate residues coordinating two Zn ions [31]. Gene names and accession numbers are as follows: mGCMa, Mouse GCM homolog 1 gene (1ODH); mGCMb, mouse GCM homolog 2 gene (EDL40969); dGCM, *Drosophila melanogaster* GCM gene (BAA10905); dGCM2, *D. melanogaster* GCM 2 gene (NP_609302); GCM_Nv, hypothetical protein gene from *Nematostella vectensis* (XP_001625315). *MuDRF* transposon sequences are deposited in Repbase (http://www.girinst.org/repbase/).
(PDF)

Figure S3 **Conservation profile for the three largest families of candidate effector proteins.** The 1,088 PST-130 candidate effectors were grouped by similarity (BLASTP, E-value≤e^−10^; Material S4). Cluster 33, 38 and 42 were the largest clusters with 16, 11 and 8 members, respectively. Sequences were aligned and analyzed with Web Logo (http://weblogo.berkeley.edu/). Arrows indicate the conserved cystein residues and the asterisk indicates the conserved FxC motif in the members of cluster 33.
(PDF)

Table S1 **Micro-synteny analysis between genes in the 20 longest PST-130 contigs and their candidate orthologs in PGTG.**
(PDF)

Table S2 **Manual annotation of genes based on comparison between PST-130 and PGTG.**
(PDF)

Text S1 **Curated sequences of PGTG peptides using a comparative approach as described in Table S2.**
(PDF)

Material S1 **Accession number and fold-coverage (coverage/median coverage) of each PST-130 contig (genomic and mitochondrial contigs are listed in separate sheets).**
(XLSX)

Material S2 **Amino acid sequences of 22,815 peptides predicted** ***ab initio*** **using geneid with parameters trained for PGTG genes.** The 20 gene models annotated using a comparative approach replaced the incorrectly predicted peptides. Gene IDs include the number of the contig where the gene was identified (first number after underscore) and the specific number of the gene on the contig (second number after underscore).
(TXT)

Material S3 **20,423 predicted PST-130 proteins with pfam accession numbers and names (** http://pfam.sanger.ac.uk/ **), GO descriptions (** http://www.blast2go.org/ **) and the most similar PGTG protein accessions and annotations (BLASTP, E-value ≤e^−5^).**
(XLSX)

Material S4 **1,088 PST-130 proteins with predicted signal peptide. Additional columns describe the grouping of these accessions based on sequence similarity (BLASTP, e-value ≤10^−10^), the availability of EST sequences from haustoria (TBLASTN, e-value ≤10^−10^) and the presence of different motifs conserved in other** ***Avr*** **genes** [**23**] **and secreted fungal proteins** [**22**] **.**
(XLSX)
