# Supplementary material for: Next Generation Sequencing Provides Rapid Access to the Genome of Puccinia striiformis f. sp. tritici, the Causal Agent of Wheat Stripe Rust
Source: PLoS One. 2011 Aug 31;6(8):e24230. doi: 10.1371/journal.pone.0024230 (PMC3164196; doi:10.1371/journal.pone.0024230)
Supplement: Figure S1 — Phylogeny of elements of the Harbinger superfamily of DNA-transposons. The conserved DDE-transposase domain (∼200 aa) was used for phylogenetic analysis using the maximum likelihood algorithm. The tree was rooted using the outgroup ISL2EU and the numbers at nodes are bootstrap values of 100 replicates (only values >50% are shown). Harbinger elements from rust are colored in purple, those from Phytophthora infestans are in red, and those from plants are in green. All sequences used are deposited in Repbase. The elements were obtained from the following species: Fungi [Puccinia striiformis f. sp. tritici (PSt), Puccinia graminis (PGr), Melampsora larici-populina (MLP, Mlarici), Allomyces macrogynus (AllMac), Ascosphaera apis (AAp), Phycomyces blakesleeanus (PB), Pleurotus ostreatus (PleOst), Talaromyces stipitatus (TSt), Tuber melanosporum (TMe)]; Plants [Arabidopsis lyrata (ALy), Fragaria vesca (FV), Malus x domestica (Mad), Medicago truncatula (Mt), Oryza sativa (OS), Populus trichocarpa (PTr), Selaginella moellendorffii (Smoe), Sorghum bicolor (SBi), Triticum aestivum (TA), Vitis vinifera (VV), Zea mays (ZM)]; Oomycetes [Phytophthora infestans (PI)]; Animals [Aedes aegypti (AA, AAe), Anopheles gambiae (AG), Branchiostoma floridae (BF), Ciona savignyi (Cis), Danio rerio (DR), Drosophila willistoni (DW), Drosophila yakuba (DYa), Gasterosteus aculeatus (GA), Hydra magnipapillata (HM), Nematostella vectensis (NV), Strongylocentrotus purpuratus (SP), Xenopus tropicalis (XT)]; Protists [Ectocarpus siliculosus (ES), Emiliania huxleyi (EmiHux), Monosiga brevicollis (MBr), Naegleria gruberi (Ngru), Thalassiosira pseudonana (TP), Trichomonas vaginalis (TV)]. (PDF) [file pone.0024230.s001.pdf]

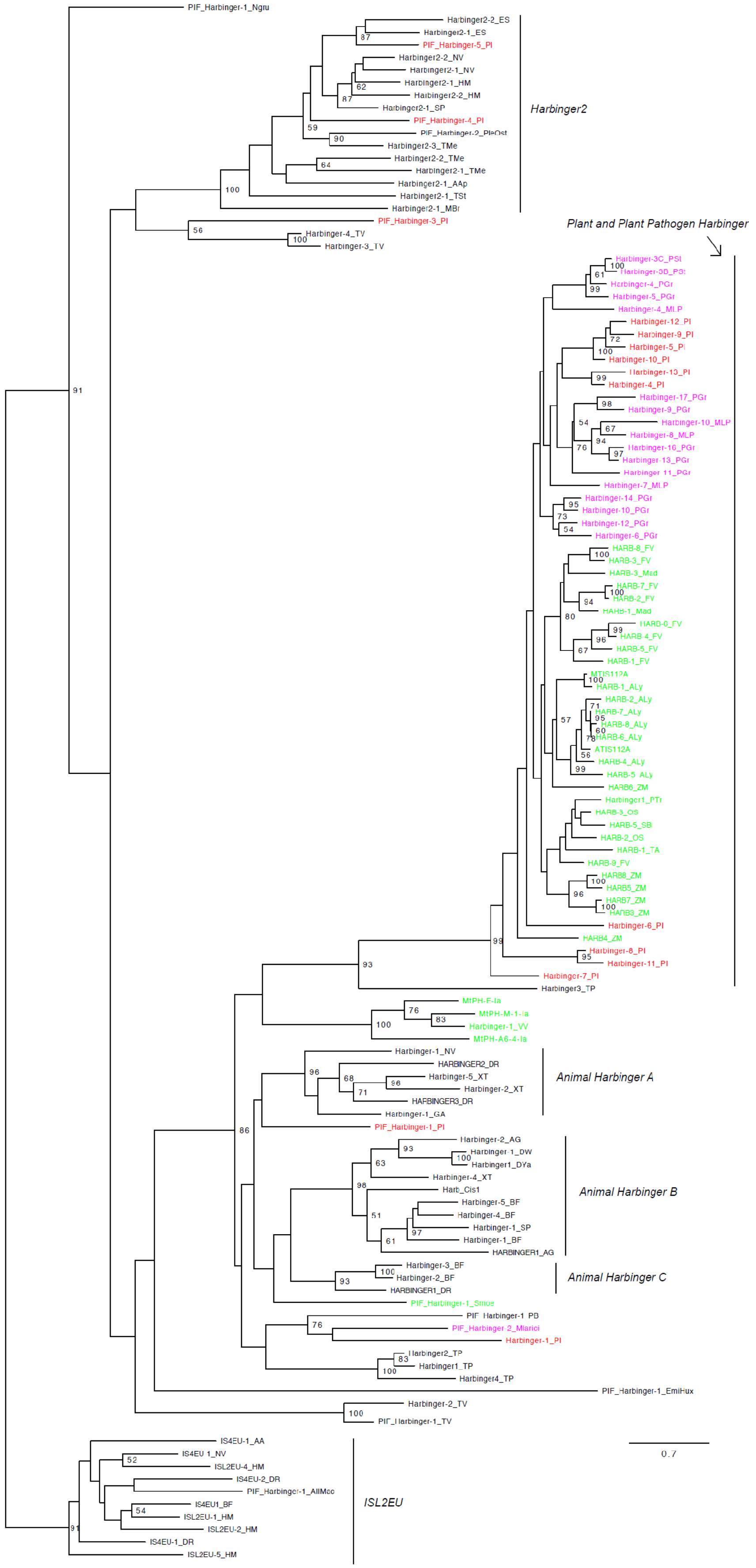

Figure S1 - Phylogeny of elements of the *Harbinger* superfamily of DNA-transposons.

The conserved DDE-transposase domain (~200 aa) was used for phylogenetic analysis using the maximum likelihood algorithm. The tree was rooted using the outgroup *ISL2EU* and the numbers at nodes are bootstrap values of 100 replicates (only values >50% are shown). *Harbinger* elements from rust are colored in purple, those from *Phytophthora infestans* are in red, and those from plants are in green. All sequences used are available in Repbase. The elements were obtained from the following species: Fungi [*Puccinia striiformis* f. sp. *tritici* (PSt), *Puccinia graminis* (PGr), *Melampsora larici-populina* (MLP, Mlarici), *Allomyces macrogynus* (AllMac), *Ascosphaera apis* (AAp), *Phycomyces blakesleeanus* (PB), *Pleurotus ostreatus* (PleOst), *Talaromyces stipitatus* (TSt), *Tuber melanosporum* (TMe)]; Plants [*Arabidopsis lyrata* (ALy), *Fragaria vesca* (FV), *Malus x domestica* (Mad), *Medicago truncatula* (Mt), *Oryza sativa* (OS), *Populus trichocarpa* (PTr), *Selaginella moellendorffii* (Smoe), *Sorghum bicolor* (SBi), *Triticum aestivum* (TA), *Vitis vinifera* (VV), *Zea mays* (ZM)]; Oomycetes [*Phytophthora infestans* (PI)]; Animals [*Aedes aegypti* (AA, AAe), *Anopheles gambiae* (AG), *Branchiostoma floridae* (BF), *Ciona savignyi* (Cis), *Danio rerio* (DR), *Drosophila willistoni* (DW), *Drosophila yakuba* (DYa), *Gasterosteus aculeatus* (GA), *Hydra magnipapillata* (HM), *Nematostella vectensis* (NV), *Strongylocentrotus purpuratus* (SP), *Xenopus tropicalis* (XT)]; Protists [*Ectocarpus siliculosus* (ES), *Emiliania huxleyi* (EmiHux), *Monosiga brevicollis* (MBr), *Naegleria gruberi* (Ngru), *Thalassiosira pseudonana* (TP), *Trichomonas vaginalis* (TV)].
