# Supplementary material for: Next Generation Sequencing Provides Rapid Access to the Genome of Puccinia striiformis f. sp. tritici, the Causal Agent of Wheat Stripe Rust
Source: PLoS One. 2011 Aug 31;6(8):e24230. doi: 10.1371/journal.pone.0024230 (PMC3164196; doi:10.1371/journal.pone.0024230)
Supplement: Figure S2 — Alignment of GCM domains coded by glial cells missing (GCM) genes and by MuDRF transposable elements. Similarity to other MuDR transposases was established after four PSI-BLAST iterations using MuDRF transposase as a query. MuDRF elements were also found in other Basidiomycota, including PGTG, M. larici-populina, Laccaria bicolor, Schizophyllum commune and Coprinopsis cinerea. Outside fungi we also identified a full-length MuDRF element in Heterolobosea (Naegleria gruberi). MuDRF elements are flanked by 9-bp TSD, characteristic for MuDR elements. Triangles indicate residues coordinating two Zn ions [31]. Gene names and accession numbers are as follows: mGCMa, Mouse GCM homolog 1 gene (1ODH); mGCMb, mouse GCM homolog 2 gene (EDL40969); dGCM, Drosophila melanogaster GCM gene (BAA10905); dGCM2, D. melanogaster GCM 2 gene (NP_609302); GCM_Nv, hypothetical protein gene from Nematostella vectensis (XP_001625315). MuDRF transposon sequences are deposited in Repbase (http://www.girinst.org/repbase/). (PDF) [file pone.0024230.s002.pdf]

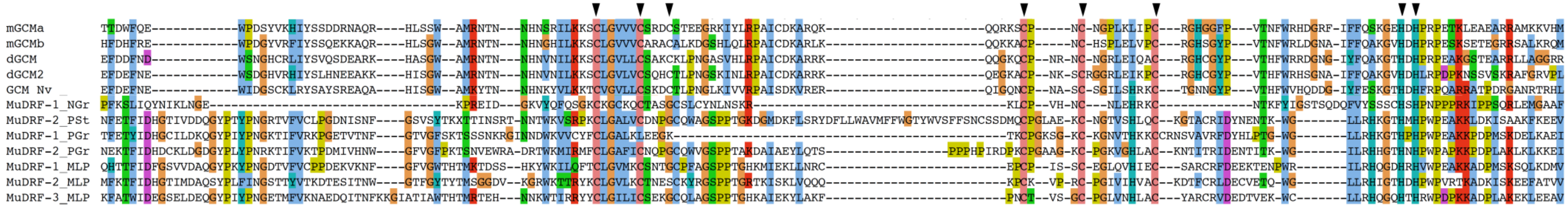

Figure S2 - Alignment of GCM domains coded by glial cells missing (GCM) genes and by *MuDRF* transposable elements.

Similarity to other *MuDR* transposases was established after four PSI-BLAST iterations using *MuDRF* transposase as a query. *MuDRF* elements were also found in other Basidiomycota, including PGTG, *M. larici-populina*, *Laccaria bicolor*, *Schizophyllum commune* and *Coprinopsis cinerea.* Outside fungi we also identified a full-length *MuDRF* element in *Heterolobosea* (*Naegleria gruberi*). *MuDRF* elements are flanked by 9-bp *TSD*, characteristic for *MuDR* elements. Triangles indicate residues coordinating two Zn ions [31]. Gene names and accession numbers are as follows: mGCMa, Mouse GCM homolog 1 gene (1ODH); mGCMb, mouse GCM homolog 2 gene (EDL40969); dGCM, *Drosophila melanogaster* GCM gene (BAA10905); dGCM2, *D. melanogaster* GCM 2 gene (NP_609302); GCM_Nv, hypothetical protein gene from *Nematostella vectensis* (XP_001625315). *MuDRF* transposon sequences are deposited in Repbase (http://www.girinst.org/repbase/).
