# Supplementary material for: Next Generation Sequencing Provides Rapid Access to the Genome of Puccinia striiformis f. sp. tritici, the Causal Agent of Wheat Stripe Rust
Source: PLoS One. 2011 Aug 31;6(8):e24230. doi: 10.1371/journal.pone.0024230 (PMC3164196; doi:10.1371/journal.pone.0024230)
Supplement: Figure S3 — Conservation profile for the three largest families of candidate effector proteins. The 1,088 PST-130 candidate effectors were grouped by similarity (BLASTP, E-value≤e−10; Material S4). Cluster 33, 38 and 42 were the largest clusters with 16, 11 and 8 members, respectively. Sequences were aligned and analyzed with Web Logo (http://weblogo.berkeley.edu/). Arrows indicate the conserved cystein residues and the asterisk indicates the conserved FxC motif in the members of cluster 33. (PDF) [file pone.0024230.s003.pdf]

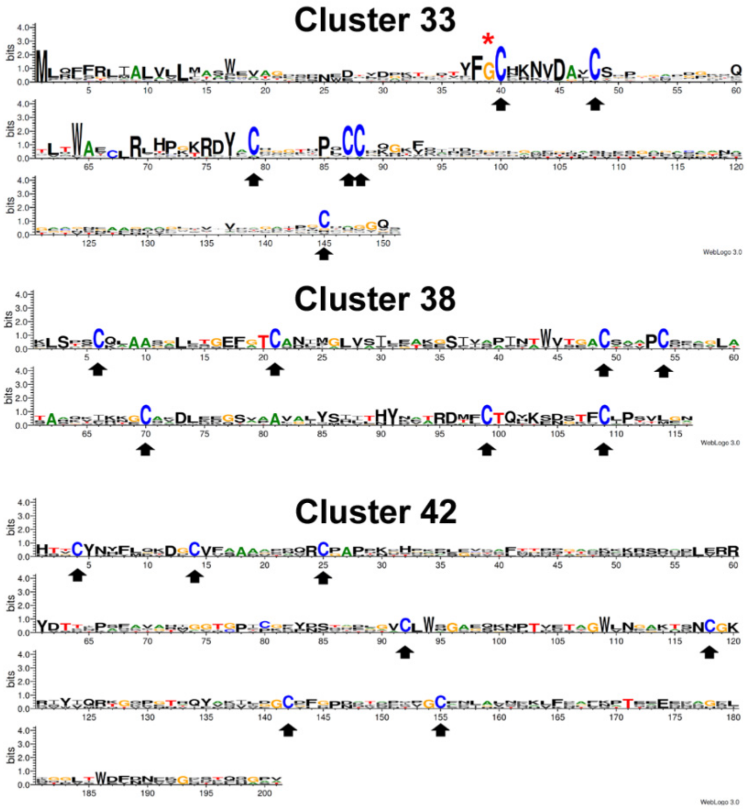

Figure S3 - Conservation profile for the three largest families of putative effector proteins.

The 1,088 PST-130 putative effectors were grouped by similarity (BLASTP, E-value≤e^{-10}; Supplementary material S5). Cluster 33, 38 and 42 were the largest clusters with 16, 11 and 8 members, respectively. Sequences were aligned and analyzed with Web Logo (http://weblogo.berkeley.edu/). Arrows indicate the conserved cystein residues and the asterisk in cluster 33 indicates the conserved FxC motif.
