# Supplementary material for: Next Generation Sequencing Provides Rapid Access to the Genome of Puccinia striiformis f. sp. tritici, the Causal Agent of Wheat Stripe Rust
Source: PLoS One. 2011 Aug 31;6(8):e24230. doi: 10.1371/journal.pone.0024230 (PMC3164196; doi:10.1371/journal.pone.0024230)
Supplement: Table S1 — Micro-synteny analysis between genes in the 20 longest PST-130 contigs and their candidate orthologs in PGTG. (PDF) [file pone.0024230.s004.pdf]

**Table S1 –** Micro-synteny analysis between genes in the 20 longest PST-130 contigs and their putative orthologs in PGTG

| **PST-130 contig** | **Length** | **PGTG contig** AACC0100 | **PST coordinates** | **% PST Contig** | **PGTG proteins (PGTG_)** [a] | **No. common genes** [b] | **Super contig No.** [c] | **Comments** |
|---|---|---|---|---|---|---|---|---|
| 8308 | 49498 | 1263 | 4656-44745 | 86% | **067**50, 52, 49, 48, 47, 46, 45, 44, 43 | 9 | 15 | 1 multigene family |
| 6952 | 39939 | 3515 | 4913-34166 | 73% | **174**75, 82, 81, 80, 79, 78 | 6 | 10 | |
| | | 3750 | 4913-34166 | 73% | **185**27**,** 31, 32, 33, 34, 35 | 6 | 17 | Duplicated PGTG region |
| 7251 | 39628 | 1311<br>1326 | 870-10776<br>25550-33655 | 45% | **068**18, 20, 22 / 55, 58 | 5 | 17 | |
| 7806 | 37324 | 1487<br>1482<br>1486 | 11-848<br>14684-17049<br>17707-22646 | 22% | **076**98, 97, 95, 93 / 83 / 82, 81, 80 | 8 | 20 | |
| 7242 | 37091 | 0045<br>0046<br>0047 | 2532-2566<br>8610-29864<br>31324-34905 | 67% | **003**98 / **004**01 ,02, 06, 07, 08, 09 / 16 | 8 | 1 | |
| 7402 | 36035 | 0221<br>0220<br>0219 | 11094-23115<br>28378-29074<br>33433-33520 | 34% | **008**79, 78, 76 /72 /68 | 5 | 3 | |
| 8617 | 35916 | 3253 | 3840-32211 | 79% | **166**84, 83, 80, 79, 78, 77, 76, 75, 74 | 9 | 74 | |
| 20459 | 35740 | 0414 | 486-30724 | 85% | **020**69, 73, 74, 77 | 4 | 5 | |
| 7057 | 34936 | 0617<br>0620<br>0623 | 3799-4591<br>19969-22905<br>28260-34382 | 50% | **037**01 / 08, 09 / 27, 28, 29 | 6 | 7 | |
| 13600 | 34906 | 2406 | 2562-13395 | 31% | **132**65, 64, 63, 62 | 4 | 43 | |
| | | 0390 | 19187-19576 | | 02861 | | 4 | Not syntenic [d] |
| | | 1580 | 20773-21157 | | 08875 | | 22 | Not syntenic |
| | | 3724<br>3768<br>3725 | 23514-26577<br>27273-29880<br>31944-33445 | 21% | **182**38,42 / 45 /48 | 4 | 103 | |
| 7101 | 34773 | 0559<br>1931 | 3731-30508<br>33328-34461 | 80% | **033**81, 82, 83, 84, 86, 87 / 88, 89 | 8 | 5 | |
| 7598 | 34231 | 2324/5 | 2399-29991 | 81% | **129**49, 50, 51, 56, 39, 57, 35, 59, 60 | 9 | 40 | |
| 8613 | 33720 | 0033<br>0034 | 27688-32436<br>5089-22558 | 66% | **002**95 / **003**13, 16, 15 | 4 | 1 | |
| 7582 | 33253 | 0329<br>0331 | 2010-12997<br>28970-31548 | 41% | **025**20, 19, 18 / 16 | 4 | 4 | |
| 17625 | 32924 | 0310<br>1583 | 11665-12077<br>14171-30709 | 52% | **089**15 / 13, 07 | 3 | 22 | |

| | | | | | | | | |
|---|---|---|---|---|---|---|---|---|
| 7330 | 32453 | 3539<br>3544<br>3550 | 2514-2794<br>2568-7365<br>17699-28388 | 49% | **180**37 / 48, 49, 54 / 59, 60, 61, 62, 64 | 9 | 92 | |
| 7170 | 32391 | 0886<br>0885 | 3425-25416<br>29853-29215 | 66% | **049**52, 50, 48, 47, 46 / 42 | 6 | 10 | |
| | | 0923 | 31023-32017 | 3% | **054**69, 68 | 2 | 11 | |
| 6894 | 31719 | 1834<br>1833 | 751-1060<br>4760-25274 | 66% | **105**80 / 69, 67, 66,64, 62, 60 | 7 | 28 | |
| 7946 | 31602 | 3551 | 2010-2684 | | **180**69 | | 92 | Not syntenic |
| | | 2278<br>2279<br>2280<br>2282 | 6272-8077<br>14342-14497<br>17354-23419<br>29588-31601 | 32% | **122**65/ 70 / 71 / 77 | 4 | 38 | |
| 7043 | 31566 | 0344 | 1094-28034 | 85% | **026**21, 14, 13, 11, 05, 04 | 6 | 4 | |

[a] Adjacent protein numbers usually correspond to adjacent annotated genes. Numbers separated by comas start with the same numbers in bold.
Most of the large PST contigs have large stretches that are syntenic to single PGTG contigs. In several cases when there are multiple PGTG contigs they correspond to the same PGTG supercontig. Most of the corresponding PGTG proteins that match a PST contig also have consecutive or close numbers, also indicating synteny. Altered orders indicate rearrangements.
[b] The number of syntenic genes includes those genes that have undergone rearrangements within the syntenic group of genes.
[c] Supercontigs are from PGTG genome assembly (01/11/2011, www.broadinstitute.org).
[d] Single gene matches in a different supercontig were considered not syntenic.
