# Supplementary material for: Next Generation Sequencing Provides Rapid Access to the Genome of Puccinia striiformis f. sp. tritici, the Causal Agent of Wheat Stripe Rust
Source: PLoS One. 2011 Aug 31;6(8):e24230. doi: 10.1371/journal.pone.0024230 (PMC3164196; doi:10.1371/journal.pone.0024230)
Supplement: Table S2 — Manual annotation of genes based on comparison between PST-130 and PGTG. (PDF) [file pone.0024230.s005.pdf]

Table S2 – Manual curation of genes based on comparison between PST-130 and PGTG.

PST-130				PGTG		
**PST gene [a]**	**No. of exons**	**Wrongly annotated exons**	**Errors found**	**PGTG gene [b]**	**Wrongly annotated exons**	**Errors found**
PST130_8439_6 (HQ698552)	4	4	Missing exon 1, truncated exon 4 , and non-existent exons 5 and 6.	PGTG_06567.2	3	Incorrect end of exon 4, and 2 non-existent exons in intergenic region. Merged with next gene.
PST130_14418_3 (HQ698553)	3	0	-	PGTG_15040.2	1	Incorrect start of exon 3.
PST130_21324_2 (HQ698554)	6	1	Extra exon at N terminus.	PGTG_07202.2	1	Incorrect exon 3, missing sequence.
PST130_15230_2 (HQ698555)	8	1	Last exon is incorrect.	PGTG_13421.2	0	-
PST130_7328_3 (HQ698556)	5	0	-	PGTG_12324.2	0	-
PST130_8439_8 (HQ698557)	2	2	Missing exon 1, incorrect start exon 2 and addition of an incorrect intron.	PGTG_06567.2	2	Missing exon 1, early start of exon 2, merged with previous gene.
PST130_10010_2 (HQ698558)	8	3	Early start of exon 6.	PGTG_05076.2	0	-
PST130_10079_1 (HQ698559)	9	3	Incorrect end of exon 1, start of exon 2 and end of exon 3.	PGTG_14939.2	0	-
PST130_10115_3 (HQ698560)	3	0	-	PGTG_13013.2	0	-
PST130_10098_3 (HQ698561)	2	0	-	PGTG_02106.2	0	-
PST130_7733_4 (JN033203)	5	5	Incorrect start codon and start of exon 2, retention of intron with stop codon and loss of exon 3, 4 and 5.	PGTG_11438.2 PGTG_11439.2	3	ORF was identified as two separate coding sequences.
PST130_11019_1	11	6	Missing part of exon 1 because on border of contig. Incorrect start of exon 2,8,9	PGTG_17808.2	2	Missing exon 4 and incorrect start of exon 5.

			and incorrect end of exon 8. Wrong stop codon.			
PST130_17625_16 (JN033204)	7	3	Incorrect start codon. Incorrect end of exon 1, 2 , 7. Incorrect stop codon.	PGTG_08907.2	1	Incorrect end of exon 5 with retention of entire intron.
PST130_7711_7 (JN033205)	4	0	-	PGTG_00569.2	0	-
PST130_7192_3 (JN033206)	17	3	Incorrect start codon. Incorrect start of exon 1, 3 and incorrect end of exon 4.	PGTG_12360.2	3	Incorrect start codon. Incorrect start of exon 15. Incorrect end of exon 13.
PST130_9446_5 (JN033207)	5	3	Incorrect start codon results in loss of exon 1,2 and incorrect start of exon 3.	PGTG_07027.2	0	-
PST130_7851_3 (JN033208)	4	1	Incorrect start of exon 3.	PGTG_02889.2	3	Incorrect start codon. Incorrect end of exon 3 result in retention of stop codon with consequent loss of exon 4.
PST130_13665_1 (JN033209)	4	2	Incorrect start codon and start of exon 2.	PGTG_14396.2	0	-
PST130_9763_2 (JN033210)	22	5	Missing exon 13. Incorrect end of exon 19 results in incorrect stop codon and loss of exon 20,21 and 22.	PGTG_11327.2	0	-
PST130_7471_3 (JN033211)	10	3	Incorrect start of exon 2. Missing exon 7. Incorrect end of exon 10.	PGTG_19538.2	2	Incorrect start of exon 8 and incorrect end of exon 10.

[a] PST gene names as in Supplementary material S3. In parenthesis GenBank accession numbers. Sequence of PST130_11019_1 is incomplete because on the edge of contig and was not deposited to GenBank.

[b] PGTG gene names as assigned in [5].
