# Supplementary material for: Next Generation Sequencing Provides Rapid Access to the Genome of Puccinia striiformis f. sp. tritici, the Causal Agent of Wheat Stripe Rust
Source: PLoS One. 2011 Aug 31;6(8):e24230. doi: 10.1371/journal.pone.0024230 (PMC3164196; doi:10.1371/journal.pone.0024230)
Supplement: Text S1 — Curated sequences of PGTG peptides using a comparative approach as described in Table S2. (PDF) [file pone.0024230.s006.pdf]

**Text S1 -** Curated sequences of PGTG peptides using a comparative approach as described in Table S2.

>PGTG_06567.2
MLHPESYTTVPSASISQAVLKSYDAIIVGAGIAGSALAFALTDPSRNRTGKSIPSVLLIERDLRQPDRIVGELLQPGGCLAVKRLGLRD
CLDEIEAVEVNGYGVYWGTDASQITQLALPYPPESVPMAWKDGALWNGKSPKGQAPRQQGRSFHHGRFVQRLRWKAHSRPTVTVLQATV
TDLIRCPKTDHVIGVTVKSAEEETVSFFAPINFIMDGCFSKFRRIIAPDGFKQPTVRSHFVGLLLQTPAPFDCIPLPGHGHVILRKKDP
AADQPVVDGELGVGPVLVYQIGTGETRMLVDVPGAKVPSISNGSLHSYLERQVGPILPRSLLETFHATLDSNDPADRLRVMPNSYLPPH
RQEGHGGVILMGDSMNMRHPLTGGGMTVALLDVEIISNLLGDLDDFEDWKAIEDRLAIWHQQRKSTSTCINVLAQALYSLFGAEDDNLE
ILKEGCFKYFELGGKRVSDPISLLSALIPSPLLLFYHFFSVAFYAIWIFTQQNGVQINKMFQILWTACVTILPVLWAEC*

>PGTG_15040.2
MNNRSVSGPRLPPGSRPKAIHIQKQIPSSSLAAITRRTLLLTIFSALFIGVYRWADAIKSRWYIFDPSELHKITLEAIERYPNSTSAVI
HHIVQSFESQPTLKPYITANPFPDPSPQAVSAYPSEWVFNNAGGAMGAMYIIHASITEYLIVFGTPIGTEGHTGRHTADDYFNILAGQQ
TAFAAGSLEAEVYRKGSVHHLRRGQVKQYKMPDGGCWALELAQGWIPPMLPFGLADSVFSTVDLITIYHTIRITAREIIRNLLAGKI*

>PGTG_07202.2
SSLIDPLIEFIGSFSTFNQILIYFLLSITSIISINIFNQLAIPKDPTTPPVVFHLFPFIGSAVSYGIDPYAFLESCRKKYGNVFTFVLL
NKKVTVALGLEGNALILNGKLSQVNAEEAYTAL
**GAP IN GENOMIC DNA SEQUENCING**
PKTQQAVKDCFKVASEITICTASATLQGPEVREGLNKSFANLYHDLDGGFTPLHFAFPNLPLPSYRRRDRAQVAMRNFYMNIIQKRRED
NREGQLGDMIDSLQGQTYKDGRPLTDKEIAHIMIALLMAGQHTSAATGSWLLLHLASRPDIVAELRQEQIEVFGKPGQTDDKELDPLDL
ERVQSPLMLACIKEVLRLHPPIHSIMRKVKSPITVPRTLASHNEDTPYIIPSSNFVLAAPGASQIDPAIWSSPHEFEPSRWLKLTSPFK
AGGGETQEEMVDYGFGMISSGANSPFLPFGAGRHRCIGEQFAYLQLSTLGATVIRNCELELVSNQFPKPDYTTMLVCPIKPRDVKFTRR
NTHS*

> PGTG_06567.2
MRIHCIGRGSIGSLLCFHLQSITPITLLLRSRQAQHRRSIPTLSIQLEQQDRTRTATGFTYEFLNKQQQQPIESLIVTTKAPHVLESLQ
RVRHRLSANSTVLLLHNGLGVVEELIETCFQEPSSRPTFVLATTSHGVYRIDKGLPGTQAGSHGRFCHAGLGDIRLGVLPNTTIRNCLE
RLRGHSNQPSSNDDSQSLQDDNPLLNPHSRTKPVLEEHLPDIEPETRSLHYTLSSLLNPLMVKELNTKWLPMGDFQTSALIKLTVNAAI
NPISALLETRNEALYRESSFESLCRQVCQEASAVFAAQAGQPFRPHHSLSAPNLQRVVNDIVLATRANISSMCSDIRTLATNRISPHKT
LSKANLNRIAASQAPIKIPNYQSLISGQEEKSVKETSTEIDYINGYICRLGSQFNVDTPLNQSLSDLIKLKSVAIKRAQVLPKLQRVNR
RLKINRPEDNPATPDQLDLFEKPENNPGSHQLDLFEKPENNHLADNQASVVDKS*

> PGTG_11438.2 - PGTG_11439.2
MDIILDLADQLILDCCWSRIFPIQLNDLKRPNPSLPLASFSKPRITTVTTQNQPNQANISYSSVFDCYFTCGSQAFQYQADRKLSITEV
VNSNLTFNFGAHHQRCLSRFGRDHIFRQSVSIFVIIFMGISFMYFFLSSLSYFFIFDHRLKKHPKFLKNQIKLEILCALKAIGPIDILA
LPLHLAQVRGHSKLYTHVSDIKGVSGFRLFKPILDFFKLTEKFQDDDSWHQLRATQPTSRLSPYPMLDNIVKRISLALRKTFHHNFGGG
WFYFIFSYFLFLYIIHRIEHHPVLYKRIHKTHHKWVIPTPFASYAFHPVDGFLQSMPYHIFVFIFPFHRFLYLFAFLFVTVWTILIHDS
ELIVGHRLEDYINGPTHHTLHHLYFNCNFGQYFTWTDKLFSTYRNPEVDDKSTLDLAQQKSTIRKNKEMVLEKDYQ

>PGTG_17808.2
MKQVIDSVIGTGGPNPPRRDEKSADQQYSEKSGAYQASNHQNLIPGKFNPDADPHTHYEFGGPWGSLGLMILFPCLMYYFFICLWCYDG
KLSRPDSLHPTEIIRWSSEFWQLIKLHTRPTWSATYLYMGLLIHQVALAWYMPGVPQEGLPIPSLNGGKLSYYCNALCSWYATLATVFV
LHSVLRVVRLGDVFDQLGHLMTIATIFGFAISLFYYVLPILQGQAVRMSGNHIYDFFMGAALSPKIGHIDVKLFAEVRIPWVLLFIIAV
AGSVKQYETIGYVTPNSLFMVYGTGLYINACAKGEECIPLTWDMAYEKWGWLLSFWNFAGVAFTYCHSVIYITNQPPSKYHFSTWSYVA
LYLTYTAAYYVFDTSNSQKARFKMENDSKSQITSRIYGFPQLSYGTLKNPKVLVVGNGDRKLLIDGWWAVCRHPNYTADFIQALCWAAC
SGTGSLIPYFYPAFFLVMILHRCTRNFERCSKKYGKSWDEYCSLVPYSFIPGVI

> PGTG_08907.2
MSRVALLLSFVGPLLLSKFLLESFERNPSFLDRLVVFCLSWPIAREIRLRLEQRDLRARASSRGALLAPLVSSPLPFGLSVLLTRLRMI
HSGSPGDVIHLFNSRVPKPIHPDQPTKVFRSRVMGVETIWTIDHDDAKYFLSTGFPNFGKSPLFKAGFRRLLGDGVFASDQRGLWAWHR
SLTRPHFVRERIADVVAMEEHSHRVATWLSTQTDLGKSVDIQDIFARYTLTVGTQHLFGRCVDSLNDLIHDRIQTGPNAADFAQNFVAA
QHWAIINSLLLHPLLISLGFRIRDRATEEVRQVVDTLVQDASLSLASQIKKNNESDSSDQAEGGTASENLLDHLLTSGCSKELVRQECL
NILLAARDTTASLLSSCIYELARDSPRKTAMWRKLKDEVERLGSGIDVTLDQVRELKYLRAVLNESLRLHPPVWANTRHAFEDDVLPSG
VFVPAGTDCRFFIREFQRNPEVWGKDAEEFDPDRWIDSRKALQVKDPFSFQPFSAGPRICLGQQFALAEASMMMIRVIEGFEGVDLDLS
DGPVGAEAPAVVLSFRGGLKVRFKK

>PGTG_12360.2
MTNNPKPSWAGPLGSTDPSRWHLQPVPDEDQAVWTYDSSPDRNLGAQSFQSKYWLSRHSKSPALPDPEGDPLQAAKNGFEFYKKLQMPD
GHWSGEFSGPLFLTPGMVIACYITKTPLAEEVKIELARRFANDQRQGQNVRDRGWGLHTAGKSTVFGTVLNYVACRLLGIDAEHTMMVR
ARATLHALGGATGIPTWGKVWLALLGVYDWEGVNPVPPELWLLPEILPLHPWRWWVHSRQVYLPISYLCGKRLQAQSDPTLDSLRKELY
TQPYESIDWPRCRNLIAKEDLYSPRHPIANGLFWILGYWEKICPSSIRNLGLNRAHELCKMEDENTDFNDLAPVNKVLNLIVCWDRYGP
ESDELRRHQLKLKNFMWMNKDGMGMSATNGSQLWDLGFITQALVESGLAKTEEPSTQDSVIRALQWIDRCQILENPKHFKSGYRHQSKG
AWPFSTKSQSYTVSDCTAEALKSVLCLQEELSYTPKLISKERLCLAVDVILSLQNPNGGFASYELIRGPSWLEYLSPAEVFGKTMIEVN
YPECTTACLTAMSLFSQYYPDYRAPEISRARQAAIKFIHSAQRDDGSWYGSWGVCFTYATMFALESLSLNNETYKNSLLVKKACRFLLD
RQMDDGGWGESFKSCEQGVYIHHQTSQVFQTAWAVLALLAAKYPEPEPIQRACRLIISRQTADGQWLDGAIEGVFNKTTSVTYPHYKFA
WSISALGKAHKRFPDVQW

>PGTG_02889.2
MAFQPKKIIVIGGEGFLGHNLVQTLHRTYPDSTISSLDLTKRFPDCKDEEPRKEQGQAENQETNNTHQFIQADLTSLDSLLEAFQQTEP

ELVFHTASPWSGSSSEICEKVNIQGTLNTIAACLKFGVQRLVYTSSAGVVFNGNDLINVDERLPVPKIGCDPYNTSKARAEAIVLEANG
KDSLLTCAIRPAGIFGPGDRQAIPGLIEVLKTRKHGVQIGGNTNLFDWTYVDNVVHAHILAAEKLDRVVPLGEFSTSLSPISKTVERRN
LLTSGSKAEEDDSSMGGTDEVSLVDLSTSSKDAESYNPTGWIQGHEGMVDQAVPAKRHRWDQWAPISTQINYPDDQVRVAGEAFFVTGG
EPVFFWDFARAVWHEYAAHSPQAKALNLDPKPRFTIVIPTFLALFLASLAQLFAKLTNSTTLFTPEKVRYTSASKYHNIEKARVVLGYE
PLIGIHEGIQKAVQWYISNESLTQSSQTSDKKID

> PGTG_19538.2
METKNPCSTGGGLHPPDTMKIPRLYQAELLEEAKKRNIIIRADTGTGKTFVALSLITWIAAQSPANHDDHRIQAFLAPTRPLAHQQAEY
IQKHCTLRVKAYTGDLQPELWNIDKWHSELNEVDVIVSTAQVSLLIFDEAHHCRKNHIYNQIMRSHYHRLAKDPTVRLPKILGLTASPI
WNYKDLERADSDIKSLQSALAAQIYEVKTHTEDVCQHNFKPNEKVVYFEPSPEFEKNSHPPWDQINQLLSLHASPKMIAAMESVSLELG
TYAHSLAVLDWLKSLLTVGASNQAMPGRLLDPNKQKQIREVIQELEELVNIDDIPETQFSSKVAVLNKILVSYKEKDNHDNFLCIVFVE
RRQHAQLLPFLLERNAQLKGFLRPTALTGHAGGNVNDLIGIKMDSRTQNKAVAKFRTGEYNLTIATSVAEEGLDFRSCRVVIRFDLITT
WKGYIQSRGRARARESDYIVMLPNGTTNKYLEFTGKEEQLKAALYNRPEDELIEEGEEEWTPQLICQLAGGKESILTYSAATSLLNDVC
QLIPPDEFLPVVAPQYEITWLGDNFQCQVTLPPMAALHPSQRTFTGLAMATKKDAKRSAAFEACKVLRELDVLNQHFLPQREGKSAQIC
DADGREIEATPLSDQVEAIIPNVYGDFRTSTEIWLHKFSFPDDSPDGFSTMGLLCARHLTVPDGLQLFDHYTDSRPLPITIEQSKRIQW
GQDDAPTNLQRLETFSRVVMQAAINRKAYEGKLYFLVAPLLRDTCEIDWNLVDTPMIPLSDTADSLRYQNTIAPIRHLHYRIFDTCEPA
GDISEASPPQSVPACPSMRDFCKKISKFHNLGHFYKVVYDLKEEQFQGELVYLETTFHVLNNLSKSESTVVQPHRILLPLKLCKGTHIP
RSMWKVFSYLPSLTRLLHDSLQATTLFKRLDFPTISLLHGIQALTPPGGGVPWDYQTLETLGDAFLKLATSVHVYLSHLKKGEGDMSHV
RSRSVDNAYLRRKAIQANLPASILSQRFRTDRFRDPQTEDGKELPNGNFSRKIPKRVLSDVVEALLGAGFLTGGIELGLKIGTALDLCF
GGTAPWSERPVNIGLESITHDALEPSTLLKCQALEEKIGYVFKEKLLLVQALTHRSANSFMTNCYEREEWLGDAVIDMWIVEHAYKRFD
HATAEELTLARAKVVSNGSLGFLALKKLGLQEIIMHASENFEQACTEAIEAIKPFAKIEEYFSTIDNLFVVFDPPKILNDVLEAIVGAV
FVDSGFNLQSAYRTLDIIFEDVIPGLSRLVARDPLSTMLRLRDQYQCAELRRISEPNPNGETKDPISVKVCRIELHGQEIASGRHKSSA
SVAEQRASLEALKVLQEPPSESPTSHSVWSTCQCKTLAVAATTLASSNLAKSKV
